# Acute liver failure in children—Is living donor liver transplantation justified?

**DOI:** 10.1371/journal.pone.0193327

**Published:** 2018-02-23

**Authors:** Marek Szymczak, Piotr Kaliciński, Grzegorz Kowalewski, Dorota Broniszczak, Małgorzata Markiewicz-Kijewska, Hor Ismail, Marek Stefanowicz, Adam Kowalski, Joanna Teisseyre, Irena Jankowska, Waldemar Patkowski

**Affiliations:** 1 Department of Pediatric Surgery & Organ Transplantation, The Children’s Memorial Health Institute, Warsaw, Poland; 2 Department of Gastroenterology, Hepatology and Immunology, The Children’s Memorial Health Institute, Warsaw, Poland; 3 Department of General, Transplant and Liver Surgery, Warsaw Medical University, Warsaw, Poland; University of Toledo, UNITED STATES

## Abstract

**Objectives:**

Living donor liver transplantation (LDLT) in patients with acute liver failure (ALF) has become an acceptable alternative to transplantation from deceased donors (DDLT). The aim of this study was to analyze outcomes of LDLT in pediatric patients with ALF based on our center’s experience.

**Material and methods:**

We enrolled 63 children (at our institution) with ALF who underwent liver transplantation between 1997 and 2016. Among them 24 (38%) underwent a LDLT and 39 (62%) received a DDLT. Retrospectively analyzed patient clinical data included: time lapse between qualification for transplantation and transplant surgery, graft characteristics, postoperative complications, long-term results post-transplantation, and living donor morbidity. Overall, we have made a comparison of clinical results between LDLT and DDLT groups.

**Results:**

Follow-up periods ranged from 12 to 182 months (median 109 months) for LDLT patients and 12 to 183 months (median 72 months) for DDLT patients. The median waiting time for a transplant was shorter in LDLT group than in DDLT group. There was not a single case of primary non-function (PNF) in the LDLT group and 20 out of 24 patients (83.3%) had good early graft function; 3 patients (12.5%) in the LDLT group died within 2 months of transplantation but there was no late mortality. In comparison, 4 out of 39 patients (10.2%) had PNF in DDLT group while 20 patients (51.2%) had good early graft function; 8 patients (20.5%) died early within 2 months and 2 patients (5.1%) died late after transplantation. The LDLT group had a shorter cold ischemia time (CIT) of 4 hours in comparison to 9.2 hours in the DDLT group (p<0.0001).

**Conclusions:**

LDLT is a lifesaving procedure for pediatric patients with ALF. Our experience showed that it may be performed with very good results, and with very low morbidity and no mortality among living donors when performed by experienced teams following strict procedures.

## Introduction

Although the first reported case of acute liver failure (ALF) was described in 1946, the definition was introduced in 1970 by Trey and Davidson [1]. Indeed, ALF is usually defined as a clinical syndrome characterized by an abrupt onset of jaundice and hepatic encephalopathy within 8 weeks after first clinical symptoms, often in the absence of any liver disease [1]. However, this generally accepted definition does not fully apply to ALF in children. Encephalopathy in children, particularly in infancy, may occur much later or not at all during ALF development. Even when encephalopathy symptoms are present, clinical ALF is often difficult to diagnose. The first definition of ALF in children was introduced by Bhaduri and Mieli-Vergani in 1996 [2]. According to this definition, it is a multisystem disorder in which there is a severe impairment of the liver function with or without encephalopathy, but with hepatocellular necrosis in children who did not have any symptoms of chronic liver disease. Overall, the outcome of liver transplantation (LT) for ALF is worse in children than in adults and especially worse when compared to the results of LT for chronic liver diseases; it is also associated with high mortality [3, 4]. Indeed, the survival of liver transplant recipients for ALF depends mainly on the urgent (hours or days) availability of a suitable donor. This is particularly difficult in pediatric patients as the possibility of harvesting a matching liver from a deceased donor (DD) is especially unpredictable.

Living Donor Liver Transplantation (LDLT) for ALF patients was first reported by Matsunami in 1992 [5]. Since then LDLT has been widely accepted after good results were first reported by Kato et al. from Japan in 1997 [6]. The aim of this study was to analyze our own experience in pediatric patients with ALF who underwent LDLT. We present clinical outcomes as well as the feasibility and safety of LDLT procedures for donors who qualified for donation usually in urgent situation.

## Material and methods

Between 1990 and 2016 there were 689 LTs performed in pediatric patients in Children’s Memorial Health Institute (CMHI), including 312 LDLTs (45%). Among 689 LT patients, 63 were children transplanted for ALF (9.1%) between 1997 and 2016: 24 (38%) underwent LDLT and 39 (62%) were transplanted with a graft from a deceased donor (DDLT). Patients were qualified for LT based on the King’s College three criteria, or specific criteria in a case of Wilson’s disease.

Retrospective analysis was performed to assess recipients and donors data:

- patients clinical status at admission and at transplantation
- total bilirubin and INR (international normalized ratio; maximum values)
- Pediatric End-Stage Liver Disease (PELD)/ Model End-Stage Liver Disease (MELD) score
- time between the recipient listing for transplantation and transplant surgery
- graft characteristics (graft to recipient weight ratio–GRWR, ABO compatibility, type of graft, cold ischemic time—CIT, optimal, suboptimal donor)
- recipient and graft postoperative and long- term follow up
- duration of living donor evaluation
- perioperative and long- term follow up of living donors (hospital stay, complications).

The alternative of a LD donation was proposed mainly to the parents of children with low body weight, in whom a chance of LT from DD was minimal, or in cases of patients with a rapidly deteriorating condition. The potential complications of donor partial hepatectomy were carefully explained. Initial screening took place in our center and consisted of an assessment of basic biochemical and virological parameters. Medical history and interview with a psychologist was carried out. Other performed tests: angio-CT, liver volumetry and accurate internal examination were performed at the center for adults, where the part of liver from LD was harvested. GRWR was estimated and considered to be adequate when between 1% and 4%.

Whenever possible the results were compared between LDLT and DDLT groups. Patient and graft survival were analyzed using the Kaplan-Meier method. For statistical analysis the log-rank (Mantel-Cox test) was used to compare survival curves. For continuous variables the Mann-Whitney test and for categorical variables Pearson's chi-squared test was used. A p-value of less than 0.05 was considered to be statistically significant. The statistical software package Graph Pad PRISM 5.0 and Statistica 10.0 were used for statistical analysis. Median values with interquartile ranges were used for numerical data.

### Ethics statement

The research project “Analysis of indications and results of LT of children with ALF” obtained an approval from the Research Ethics Committee at the Children's Memorial Health Institute, Warsaw, for the realization of our project and included a retrospective analysis of indicated material (Approval no 5/KBE/2009). At the moment of admission to our hospital a conscious consent allowing the anonymous use of patients’ medical data was signed by Guardians/Parents of children of age ≤16 years. This form of voluntary consent is a standard procedure approved by the ethics committee in our institution and is applicable to all patients treated in our institute. We have obtained all the patients’ data from patients’ medical history documentation and subsequently created an anonymous database as a starting point for our analysis. All the data were anonymized/de-identified prior to analysis. Our research was conducted in accordance to the principles expressed in the Declaration of Helsinki.

## Results

### Recipients

Among 69 children with ALF, 16 girls and 8 boys were transplanted with liver grafts from LD: their age ranged from one month to 15 years (median 4.0 years); their body weight ranged from 3.1 kg to 46.7 kg (median 19.5 kg). The causes of ALF in our 24 patients are listed in Table 1.

**Table 1:** The causes of ALF in children transplanted with LDLT and DDLT.

Etiology	LDLT	%	DDLT	%
	No of patients		No of patients	
Unknown	10	41.6	12	30.6
Mushroom poisoning	4	16.6	6	15.4
Paracetamol toxicity	1	4.2	1	2.6
Toxic injury	1	4.2	3	7.7
Iron poisoning	1	4.2	0	0
Acute AIH	1	4.2	1	2.6
Wilson’s disease	3	12.5	12	30.8
HBV hepatitis	1	4.2	2	5.1
HAV hepatitis	0	0	1	2.6
Neonatal hemochromatosis	1	4.2	0	0
Mitochondrial cytopathy	1	4.2	1	2.6
**Total**	**24**	**100**	**39**	**100**

AIH, autoimmune hepatitis; HBV, hepatitis B virus; HAV, hepatitis A virus

Time between the listing for transplantation and transplant surgery was between 11 and 170 hours (median 25 hours) in patients who received LDLT and between 10 and 168 hours (median 61 hours) in patients transplanted with DDLT. Encephalopathy higher than stage II at the time of hospital admission was observed in 7 (29.1%) patients receiving a LDLT and in 7 (17.9%) patients receiving a DDLT. The number of patients with stages III/IV of encephalopathy immediately prior to transplantation was 15 patients in the LDLT group (62.5%) and 25 patients in the DDLT group (64.1%). Mechanical ventilation at the time of admission was necessary in 4 recipients of LDLT (16.6%) and in 3 recipients of DDLT (7.6%). During pre-transplant treatment the number of patients on mechanical ventilation increased from 4 to 19 (79.1%) among LDLT recipients and from 3 to 24 (61.5%) among DDLT recipients. Renal failure occurred in 3 patients (12.5%) of the LDLT group and in 4 patients (10.2%) of the DDLT group at the time of admission and in 5 patients (20.8%) of the LDLT group and in 10 patients (25.6%) of the LDLT group after transplantation. Altogether 17 patients receiving a LDLT (70.8%) and 26 patients receiving a DDLT (66.6%) underwent hemodialysis (HD) or liver support albumin dialysis—molecular adsorbent recirculating system (MARS) -before transplantation. Hemodynamic failure at admission, requiring catecholamine support, was observed in 3 patients (12.5%) in the LDLT group and in one patient (2.6%) in the DDLT group. After transplantation the hemodynamic support was necessary in 12 patients (50%) of LDLT and 24 patients (61.5%) of DDLT. The LDLT group was characterized by a lower total bilirubin concentration and comparable INR levels. The PELD score in patients from the DDLT group ranged from 17 to 52 (median 36), and from 15 to 45 (median 32) in the LDLT group. The MELD value ranged from 15 to 33 (median 24) in recipients of a LD graft and from 24 to 53 (median 36) in recipients of a DD graft. None of the above results were statistically significant between LDLT and DDLT groups (p = NS) except the total bilirubin concentration (p = 0.0308; Table 2).

**Table 2:** Selected clinical data of LDLT and DDLT patients.

Clinical data/status		LDLT	DDLT	p value
Encephalopathy greater than II degree	at admission	7/24 (29.1%)	7/39 (17.9%)	p = .2983
	before LT	15/24 (62.5%)	25/39 (64.1%)	p = .8979
Mechanical ventilation	at admission	4/24 (16.6%)	3/39 (7.6%)	p = .2710
	before LT	19/24 (79.1%)	24/39 (61.5%)	p = .1444
Renal failure	at admission	3/24 (12.5%)	4/39 (10.29%)	p = .7832
	before LT	5/24 (20.8%)	10/39 (25.6%)	p = .6635
Hemodynamic support	at admission	3/24 (12.5%)	1/39 (2.6%)	p = .1163
	before LT	12/24 (50.0%)	22/39 (56.4%)	p = .6201
HD, MARS	at admission	0	0	(-)
	before LT	17/24 (70.8%)	26/39 (66.6%)	p = .7301
Total bilirubin (mg/dl)	before LT	med. 15 (1.7–48.9)	med. 23.6 (2.8–73.9)	**p = .0308**
INR	before LT	med. 4.7 (2.02–10.0)	med. 4.8 (2.5–11)	p = .3516
PELD score (< 12 yrs)	before LT	med. 32 (15–45)	med. 36 (17–52)	p = .0645
MELD score (12–18 yrs)	before LT	med. 24 (15–33)	med. 36 (24–53)	p = .0758
Time from listing to transplantation (hrs)		med. 25 (11–170)	med. 61 (10–168)	p = .3849

HD, hemodialysis; MARS, Molecular Adsorbent Recirculating System; INR, international normalized ratio; PELD, Pediatric End-Stage Liver Disease; MELD, Model End-Stage Liver Disease; LT, liver transplantation

### Live donor analysis

After initial screening, 26 potential LDs were evaluated and 24 LDs (92.3%) were accepted after the full examination process. Only 2 LDs were excluded due to medical contraindications. The donors’ ages ranged between 21 to 54 years (median 34 years) and body weight was between 44.6 to 92 kg (median 63 kg). Mothers were donors in 14 cases whereas fathers in 8 cases and grandmothers in 2 cases. The detailed donor evaluation was completed within 24 hours in all cases: GRWR ranged from 0.86 to 8.7 (median 1.85) and weight of the graft from 189 g to 617 g (median 319 g). In one case the donor graft was further reduced to a monosegment (segment III) before transplantation. Bisegmental grafts (segments II and III) were transplanted in 15 patients, left liver (segments II, III and IV) in 7 patients and right liver (segments V, VI, VII, and VIII) in one patient. The donor and recipient blood types were identical in 14 patients (58.3%), non-identical but compatible in 5 patients (20.8%), and incompatible in 5 patients (20.8%). The CIT in patients for LDLT ranged between 2.4 and 5.4 hours (median 4.0).

### Deceased donor analysis

The deceased donors’ ages ranged from 15 to 58 years (median 38.5 years) and body weight from 50 to 100 kg (median 70 kg). Only 10 out of 39 DD (25.6%) were categorized as optimal and only 1 was under 18 years old. The majority of 29 DD grafts (74.4%) were harvested from suboptimal donors due to various reasons. The whole liver was transplanted in 31 patients (79.5%), left lobe in 5 patients (12.8%), and left lateral lobe in 3 patients (7.7%). The donor and recipient blood types were identical in 15 patients (38.5%), non-identical but compatible in 9 patients (23%), and incompatible in 15 patients (38.5%). The CIT for DDLT ranged between 5 hours to 15 hours (median 9.2 hours) and was significantly longer than in LDLT (p<0.0001; Table 3).

**Table 3:** ABO compatibility and cold ischemia time (CIT)–comparison between living and deceased donor transplantation in ALF patients.

		LDLT	DDLT	p value
**ABO compatibility**	**Identical**	14/24 (58.3%)	15/39 (38.5%)	p = .1244
	**compatible, non-identical**	5/24 (20.8%)	9/39 (23.0%)	p = .8352
	**incompatible**	5/24 (20.8%)	15/39 (38.5%)	p = .1444
**CIT**		median 4.0 hours	median 9.2 hours	**p < .00001**
		(2.4–5.4)	(5–15))	

### Results of transplantation and follow-up of recipients

Follow-up periods for patients who received a LDLT ranged from 1 to 15 years (median 6 years) and also from 1 to 15 years (median 9 years) in patients who received a DDLT. There was not a single case of primary non-function (PNF) in patients after LDLT and early graft function was good in 20 out of 24 patients (83.3%). In comparison, PNF occurred in 4 recipients (10.2%) among recipients of a DDLT. Whereas, good early graft function was only present in 20 patients (51.2%). Consequently, LDLT always performed better than DDLT.

Three patients out of 24 with a LDLT (12.5%) died within 2 months after transplantation. The causes of death were listed as multi-organ failure (MOF) in 2 patients and neurological complications in one patient. Eight recipients out of 39 (20.5%) died in the early postoperative period after DDLT. The causes of death were listed as: severe post-reperfusion syndrome in onf patient; MOF in two patients; circulatory failure in 3 patients; and, neurological complications in 2 patients. All early deaths occurred within 37 post-operative days. Early mortality after transplantation in patients with ALF was higher in the DDLT group than in the LDLT group. However, this difference was not statistically significant (p = 0.4159). While there was no late mortality observed in the LDLT group, 2 patients died in DDLT group: one within 12 months after transplantation because of sepsis and one within 5 months because of hemophagocytic syndrome. (Table 4)

**Table 4:** Summary of outcomes of patients and grafts after LDLT and DDLT.

Outcome	LDLT	DDLT	p value
**Early mortality**	3/24 (12.5%)	8/39 (20.5%)	p = .4159
**Late mortality**	0	2/39 (5.1%)	p = .2596
**Re-transplantation**	2/24 (8.3%)	7/39 (17.9%)	p = .2895
**Actual patient survival**	20/24 (83.3%)	29/39 (74.3%)	p = .4054
**Actual graft survival**	19/24 (79.1%)	21/39 (53.8%)	**p = .0427**

Early mortality–the total number of deaths ≤ 60 days after LT

Late mortality–the total number of deaths > 60 days after LT

Actual patient survival–the number of patients surviving from the transplant date to the last follow-up

Actual graft survival–the number of patients with functioning transplanted liver grafts to the last follow-up

**Fig 1:**
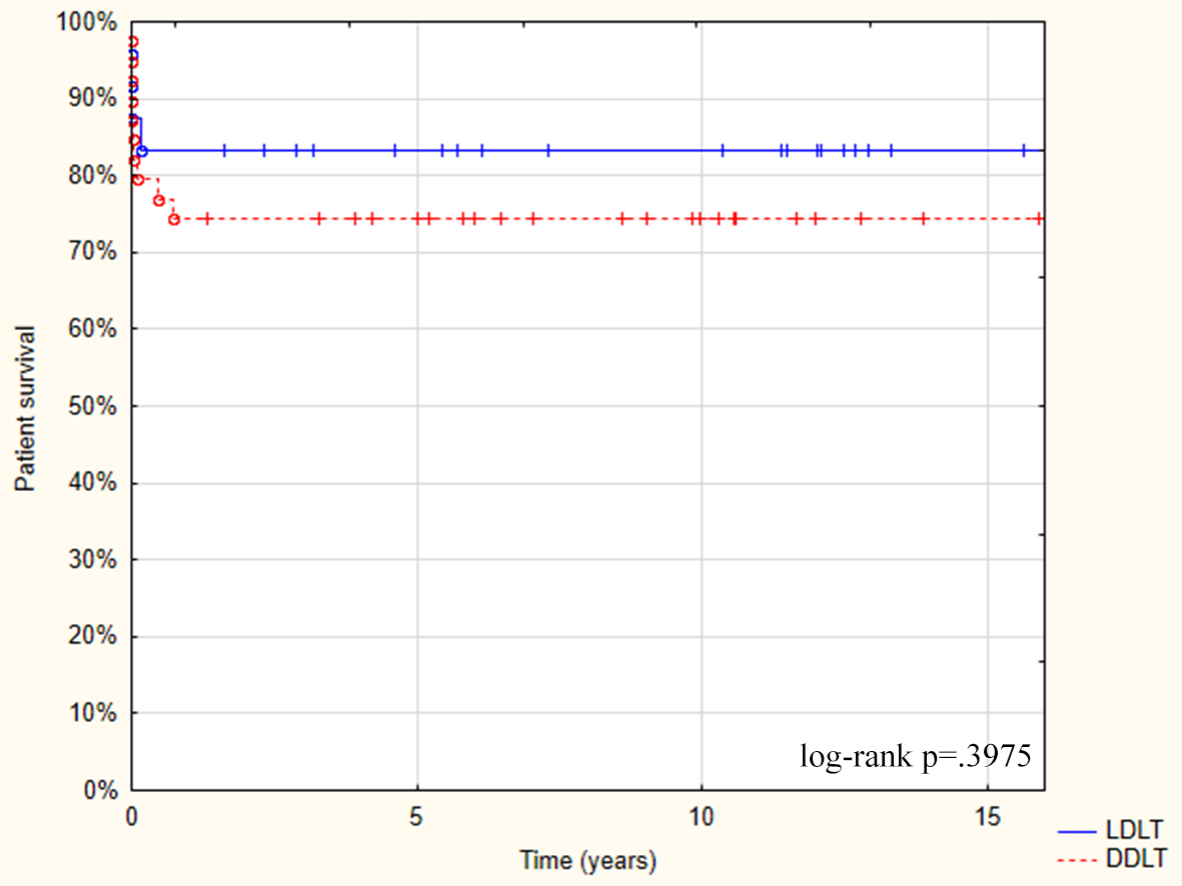
Patients’ survival after LDLT and DDLT in the ALF group with the Kaplan-Meier comparison.

The actual patients’ survival was 83.3% after LDLT and 74.3% after DDLT. However, the Kaplan-Meier comparison did not reach statistical significance (Fig 1).

**Fig 2:**
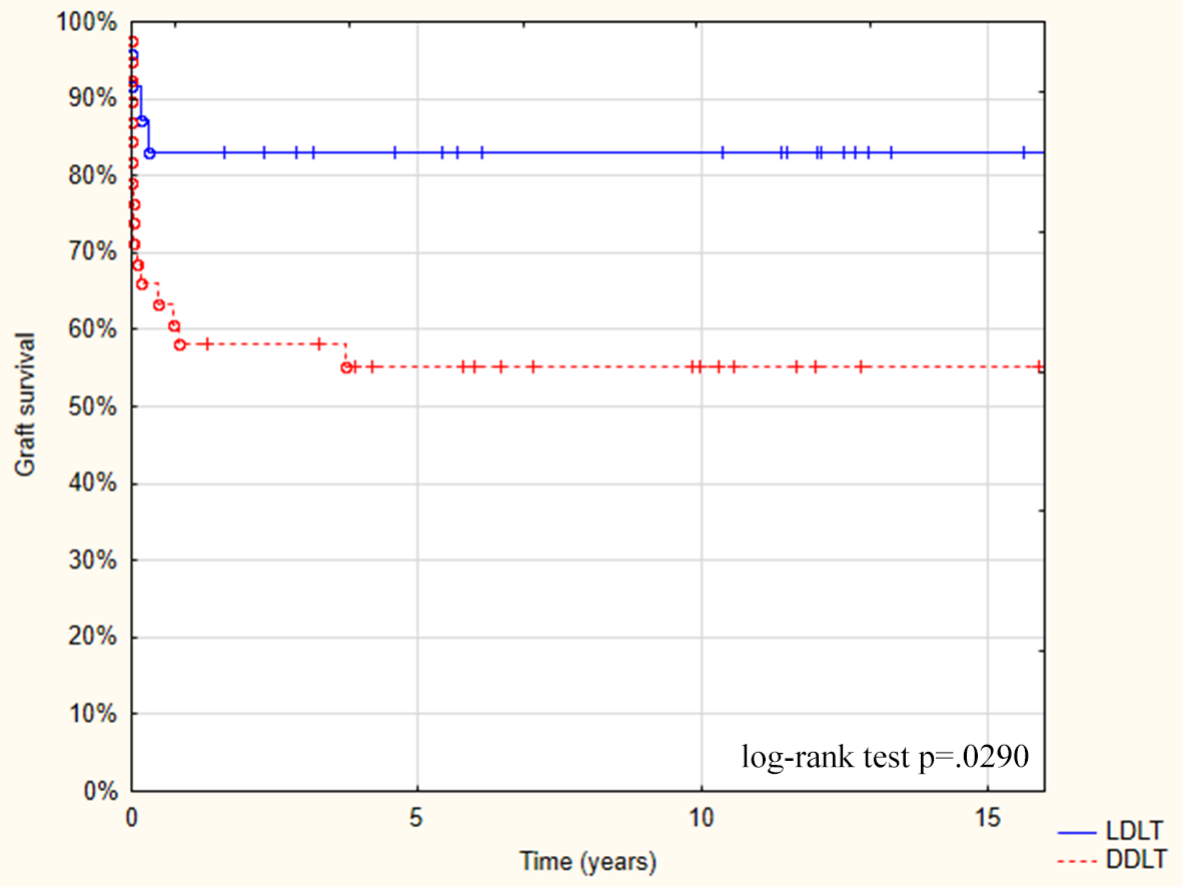
Graft survival after LDLT and DDLT in ALF groups as evaluated by the Kaplan-Meier comparison.

Two patients (8.3%) after LDLT required late re-transplantation: the first on day 58 because of biliary complications and the second on day 157 because of the vascular complications. The following complications occurred in 7 out of 39 DDLT patients (17.9%) requiring re-transplantation: 4 patients required early re-transplantation because of PNF and 3 patients required late re-transplantation because of biliary complications and/or liver fibrosis occurring at 2 months, 2 years, and 4 years. The actual graft survival for this re-transplanted group was 79.1% for LDLT and 53.8% for DDLT (p = 0.03; Fig 2).

Symptoms of permanent major neurological sequelae were found in two patients (8.3%) after LDLT and in four patients (10.2%) after DDLT. The detailed clinical outcome for ALF groups after LDLT and DDLT is presented in Table 4.

Follow-up of living donors: a thorough evaluation of liver donor surgery, and living donors was performed. The center performing harvesting from live donors has extensive experience in adult liver surgery and LT. There was no need for blood transfusion during or after operations. None of the living donors required reoperation due to postoperative complications. The hospitalization time due to living donation did not exceed 7–8 days in all cases. All donors are alive and no serious early or late complications were observed.

## Discussion

In recent years, there has been significant progress in the treatment of ALF. This progress is mainly attributed to dramatic improvements in the quality of medical care, especially in intensive care techniques, including multiple excellent extracorporeal procedures such as hemodialysis and albumin-dialysis. Despite these undisputable successes in patients’ management, the emergency LT (LDLT or DDLT) remains as the only life-saving procedure for patients with ALF. In fact, all of the improved emergency level procedures only help to prolong patients’ temporary survival while awaiting LT [7]. ALF almost always requires urgent LT as these patients deteriorate very rapidly and often end up with MOF. The clinical management of ALF patients requires knowledge, experience, extraordinary accuracy, consistency, and the ability to make the right decisions under pressure to save patients’ lives. Since ALF is a multiorgan disease with very unpredictable outcomes the deterioration of patients' condition can occur rapidly in a matter of hours or days and therefore the waiting time for LT is limited [8]. This is especially important while facing organ shortage in a pediatric population. [9] Independently of the best efforts, many patients with ALF die shortly before LT or are disqualified from LT because of existing contraindications [10]. Despite improvements in organ allocation, the mortality of patients with ALF who are waiting for LT is significantly higher than among patients who are waiting for a donor for any other reason. Moreover, the long-term results of LT are worse for recipients with ALF in comparison to recipients with any chronic liver insufficiency, despite clear progress in the intensive care management prior and after LT [7,11,12].

While dealing with ALF patients, one has to remember that meeting the strict requirements for being qualified for LT may change very fast and therefore these patients start a race against the possible irreversible damage inflicted to the central nervous system. Especially in patients with ALF, the scarcity of time becomes very critical for successful LT, as the risk of death in this group reaches up to 90% [7,11, 12]. Moreover, several factors have a significant effect on the survival of both the patient and graft itself, including the quality of the harvested organ, blood group compatibility, and graft-recipient size match. The most optimal but rarely achievable outcome would be to perform a good quality and size matched LT shortly after the patient’s introduction onto the waiting list. Only a well-functioning transplant provides a real chance for patient’s survival. However, in most cases there is always a dilemma between accepting a suboptimal quality donor and the possible worsening of the patient's condition or even death while waiting for a better donor. Pediatric transplantation deals with additional problems regarding mainly graft-recipient size match and organ quality, which only add to the complexity of existing problems and the reality of severe organ shortage. All these factors accumulate into the pressure to use available organs even of suboptimal quality. This is particularly difficult in the decision about using ABO-incompatible organs, split-organs, and organs from marginal donors. Consequently, a live donor is a vital alternative for the LT often guaranteeing a much better quality liver with a relatively short CIT [12]. Although LT from a LD is a recognized and accepted treatment for end-stage liver disease in children, it remains controversial in patients with ALF [13]. On one hand, objections mainly concentrate on the unusually short time for the precise medical and psychological evaluation of potential donors. Furthermore, LD for children are often emotionally attached, which according to some authors, may increase the complication risk of the graft harvest and transplantation procedures [14, 15]. On the other hand, in most cases this is the only chance for saving the child’s life suffering from ALF. This remains especially true in children under the age of 2 years who have very little chance of survival without an immediate LT and for whom the chance of receiving a good quality LT in a short time is very low [16].

Patients with ALF are prone to complications after LT. Because of the fast progress of ALF symptoms and a possible deterioration into MOF, there is a tremendous risk for these undesirable consequences occurring after LT from a marginal donor. These patients are most likely to have initial poor graft function or even PNF. In fact, it was reported that PNF occurred in 16% of ALF patients which was the highest complication rate of any group of LT patients [17]. In our experience, there was not a single case of PNF among our patients after LDLT and early graft function was evaluated as good in the vast majority of patients (80.4%). Among the DDLT group PNF occurred in 4 recipients (10.2%) while early graft function was evaluated as good in 20 patients (51.2%). Thus, there was a noticeable difference between LDLT and DDLT groups.

An important problem in LDLT in ALF cases is the need for rapid and efficient evaluation of potential live donors. The benefits for an ALF patient must be carefully weighed against the risk posed to a potential living donor. Based on available data, the risk of complications is small but nonetheless exists [18]. It is also possible that a parent who is highly motivated to save her/his own child’s life may intentionally conceal significant medical contraindications. Therefore, the standard for donor evaluation should be as rigorous as for any other planned live donation, but must be performed as fast as possible. The absolute rule for the LDLT program should be the safety of donors [19]. Donor examination should be minimally invasive, but must collect the essential information about donor health, liver anatomy, and organ size. Donor evaluation for ALF should include the same elements as in any planned LDLT. In particular, it should include computerized volumetric evaluation of the donor's liver, allowing for an adequate graft-recipient size match (GRWR) between 1% and 4% [20]. For the very small recipient, the reduction of bi-segmental grafts was performed on the back table when the GRWR value exceeded 5%. Reviewing data from the most experienced transplant centers, including our own observations, a 24-hour period proved to be sufficient for full assessment of the donor. This was confirmed by reports evaluating the time of examination ranging between 18 and 48 hours as sufficient to fully assess donors for their psychological and physical health [21, 22]. In our experience, no serious complications were observed in any of living donors. The harvesting procedure from live donors and transplantation to patients can be safely performed without or with very low morbidity. In our opinion, both procedures (donor harvesting and recipient LT) should be performed by very experienced multidisciplinary teams. Living donor surgery as well as the transplantation of LD grafts proved to be technically demanding and burdened with the possibility of more frequent vascular and biliary complications in recipients [20,23, 24]. Overall, a LT from a LD brings tangible benefits in terms of shortening waiting time for organ transplants, improved organ quality, and shortening of CIT. Complications resulting from organ preservation including PNF are minimized due to the short duration of CIT. All these factors are of great importance in patients with ALF and contribute to the fast recovery of patients and a good survival of grafts. In some reports, DDLT achieved a median time from listing to LT ranging from 1 to 3 days [25]. In our series, median time from listing to LT was 25 hours in the LDLT group which was shorter in comparison to 61 hours in the DDLT group, but did not reach statistical significance because of outliers (p = 0.3849). Better graft quality in the LDLT group was also evident, as 29 grafts in the DDLT group (74.4%) were harvested from DD who were of sub-optimal quality and two of them developed PNF symptoms. In contrast, patients after LDLT had much better early graft function and none of them had PNF symptoms. There are limited data in the literature about ALF patients treated with LDLT. The survival of pediatric patients with ALF after LDLT is comparable, or even better, in comparison with patients after DDLT [12, 26] ranging between 60% and 75% [7, 23, 27]. However, clinical outcomes of LDLT after ALF are worse than those in cases performed as planned non-urgent LDLT [7].

In our analysis, the actual survival rate in pediatric patients with ALF was 83.3% in LDLT group and 74.3% in DDLT group. The Kaplan-Meier comparison showed that the difference was not statistically significant (p = 0.3975), probably because of a relatively small group of patients. High mortality in ALF patients is mainly attributed to the advanced encephalopathy, toxic edema, hypoxia, and MOF, including renal failure [7, 28]. We suggest that LDLT has beneficial effects on the neurologic outcomes in our group of patients. The permanent major neurological sequelae was found only in two recipients in the LDLT group (8.3%) compared to four recipients (10.0%) in the DDLT group. The shortage of time available for treatment of ALF patients often forces the use of sub-optimal organs, including ABO-incompatible organs [11]. The use of blood group mismatched donors in LT remains controversial and is associated with the higher risk of acute rejection and other possible complications [27]. The same group reported that the blood group incompatibility resulted in worse long term outcomes including complications such as chronic bile duct injury [27]. Based on our experience LT from an AB0-incompatible donor is justified for patients requiring an urgent LT when a compatible organ is unavailable and time is running out. However, this approach should not be considered as the first option as the risk of worse outcomes is higher [27, 29, 30]. In our series, ABO incompatibility was less frequent in LDLT than in DDLT (20.8% vs. 38.5%). Overall, centers using LDLT for ALF adult patients have observed up to a 2-fold drop in the mortality rate of patients on the waiting list [31, 32, 33]. These results show significant benefits from LDLT in ALF patients.

In conclusion, our results showed that LDLT is a lifesaving procedure for pediatric patients with ALF. Our experience showed that the LDLT program for children with ALF has very good results with very low morbidity among LDs. Our recommendation is that all operations need to be performed by very experienced teams with strict procedural rules.

## Abbreviations

AIH: autoimmune hepatitis
ALF: acute liver failure
CIT: cold ischemic time
CMHI: Children’s Memorial Health Institute
DD: deceased donor
DDLT: deceased donor liver transplantation
GRWR: graft to recipient weight ratio
HD: hemodialysis
INR: international normalized ratio
LD: living donor
LDLT: living donor liver transplantation
LT: liver transplantation
MARS: Molecular Adsorbent Recirculating System
MELD: Model End-Stage Liver Disease
MOF: multiple organ failure
PELD: Pediatric End-Stage Liver Disease
PNF: primary non-function
